# Role of Vitamin C on methotrexate-induced nephrotoxicity in psoriasis context: A preclinical assessment

**DOI:** 10.1016/j.toxrep.2024.101782

**Published:** 2024-10-21

**Authors:** Elodia-Nataly Díaz-de-la-Cruz, Grecia-Elena Hurtado-Nuñez, Sandra-Guadalupe Sánchez-Ceja, Luz Torner, María-Carmen Bartolomé-Camacho, Walter-Ángel Trujillo-Rangel, Martha-Estrella García-Pérez

**Affiliations:** a Facultad de Químico-Farmacobiología, Universidad Michoacana de San Nicolás de Hidalgo, Morelia, Michoacán, Mexico; b Centro de Investigación Biomédica de Michoacán, Morelia, Michoacán, Mexico; c Centro Universitario de Tonalá. Universidad de Guadalajara, Guadalajara, Jalisco, Mexico

**Keywords:** Ascorbic acid, Kidney, Methotrexate, Necrosis, Oxidative stress, Psoriasis

## Abstract

Methotrexate (MTX) is the most prescribed drug for systemic treatment of psoriasis. However, its clinical use is limited by its nephrotoxicity, which antioxidants can attenuate. This study evaluates the impact of vitamin C (vitC), a well-known antioxidant, on nephrotoxicity induced by high MTX doses in the context of psoriasis. To achieve this purpose, the kidney injury triggered by acute MTX exposure was established in an imiquimod-induced psoriasis-like mouse model. Mice were randomly divided into six groups: group 1 (control); group 2 (Imiquimod, IMQ), group 3 (IMQ+vitC 175 mg/kg/day); group 4 (MTX 20 mg/kg i.p); group 5 (IMQ+MTX 20 mg/kg) and group 6 (IMQ+MTX 20 mg/kg + vitC 175 mg/kg/day). The effects of these treatments were determined by considering the evolution of IMQ-induced skin lesions and serum creatinine levels. Moreover, histopathological analysis, lipid peroxidation, oxidative stress, and TNF-α production were determined in kidney tissue. Results showed that vitC attenuates renal damage in the context of IMQ-induced psoriasis. However, the opposite occurs when administered with IMQ+MTX, worsening skin psoriasis lesions and exacerbating acute renal tubular necrosis and oxidative DNA damage. These results establish new clues about the MTX-induced nephrotoxicity in the psoriasis context and the putative protective effects of vitC. It suggests that vitC supplementation could help attenuate the renal damage promoted by the psoriatic pathological environment. However, it should be avoided in psoriasis patients with renal dysfunction treated with MTX.

## HIGHLIGHTS

• Renal injury promoted by Methotrexate increased in psoriasis context
• Vitamin C showed renoprotective effects by modulating kidney damage in psoriasis
• Vitamin C exacerbated renal injury triggered by MTX in psoriasis environment

## Graphical Abstract

**Figure:**
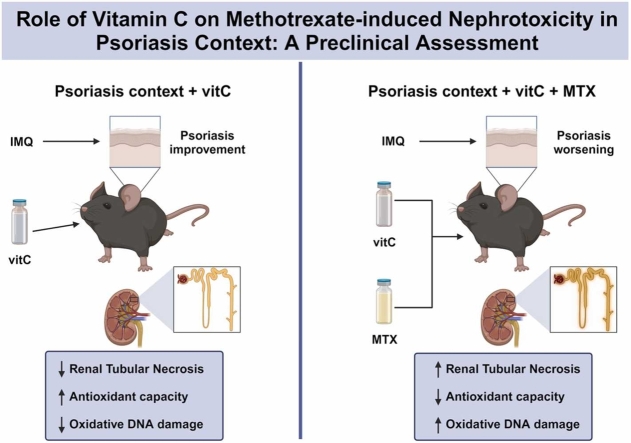

## 1 Introduction

Psoriasis is an autoimmune inflammatory disease affecting around 3 % of the world's population. It is characterized by erythro-squamous skin lesions, which have a profound negative psychosocial impact on a patient's quality of life [1].

One of the most used antipsoriatic drugs is Methotrexate (MTX) (4-amino-N10-methylpteroyl glutamic acid), an analog of aminopterin, a folic acid antagonist. It is considered an anti-inflammatory, antiproliferative, and immunosuppressive agent whose mechanism of action is based on the competitive inhibition of dihydrofolate reductase and the decrease of the synthesis of deoxythymidylic acid, which is necessary for DNA replication. Consequently, MTX can control psoriasis through the inhibition of replication of immune cells and keratinocytes [2], [3].

As a first-line antipsoriatic drug, MTX is used both by oral and subcutaneous administration at low doses (10–25 mg/week) [4], [5]. However, its clinical use can be limited by its toxicity, which provokes treatment withdrawal in 20–30 % of patients [6], mainly during long-term antipsoriatic therapies [7], [8]. The most reported adverse events related to MTX at low doses are gastrointestinal infections and hepatobiliary disorders [8], [9]. However, to a lesser extent, pneumonitis can be found in patients [7], [8]. Although less frequently, at low MTX doses, nephrotoxicity can also occur with fatal consequences [8], [10].

Psoriasis patients may also be at risk from high MTX exposure (doses greater than 500 mg/m^2^), mainly those having comorbidities such as hematologic malignancies closely related to moderate-to-severe psoriasis [11], [12], [13]. Additionally, high MTX exposition can be faced by psoriasis patients during accidental overdosing [10], [14], [15]. While gastrointestinal disorders and hepatotoxicity are common at low MTX doses, nephrotoxicity is a concern at high exposure, where acute kidney injury occurs in 2–12 % of patients [16].

Nephrotoxicity triggered by MTX is characterized by MTX tubular crystallization, increases in creatinine, and decreased kidney function [16]. The deleterious renal effects of MTX alter its renal clearance, leading to increased MTX toxic exposure and worsening kidney function, thereby increasing MTX-induced non-renal toxicity (myelosuppression, hepatotoxicity, mucositis, and skin injury) [16]. The mechanism by which MTX induces nephrotoxicity has not yet been fully understood. However, it is known that one of the factors contributing to renal dysfunction is oxidative stress (OS) [17], [18]. MTX provokes an increase of reactive oxygen species (ROS) in kidneys, accompanied by lipid peroxidation and a depletion of renal glutathione, thus impairing antioxidant defenses. Consequently, mitochondrial dysfunction is triggered, energy crisis, and renal failure [18].

Previous research has shown that psoriasis patients are at higher risk of developing kidney diseases than healthy individuals, although the pathological relationship between psoriasis and renal disorders remains debatable [19], [20], [21], [22]. Psoriasis is a disease characterized by OS, vascular dysfunction, and systemic inflammation [22], [23]. Accordingly, it is hypothesized that these conditions not only favor the development of kidney diseases but could also promote the induction of kidney damage from intrinsically nephrotoxic drugs such as MTX.

As OS is a relevant factor driving MTX-induced kidney injury, the use of antioxidant substances such as vitamin B12, curcumin, rosmarinic acid, naringin, and quercetin has been proposed to reduce the nephrotoxicity triggered by this drug [24], [25], [26], [27], [28]. Vitamin C (vitC, ascorbic acid) is a recognized antioxidant molecule, present in the human body (around 20 mg/kg) and widely available in drug stores, which not only acts by capturing ROS but also interacts as a co-enzyme in oxidative pathways [29]. Although previous studies have demonstrated that vitC modulates OS and nephrotoxicity induced by MTX in rodents [30], [31], its effects on acute kidney injury triggered by this drug in the psoriasis environment are unknown. This limits the establishment of strategies to modulate MTX-induced acute kidney injury in psoriasis patients.

This research aimed to evaluate the effect of vitC on nephrotoxicity induced by high MTX doses in psoriasis. Consequently, the kidney injury triggered by MTX was established for the first time in a murine model of psoriasis induced by imiquimod (IMQ) [32]. This study establishes new clues about the MTX-induced nephrotoxicity in psoriasis and improves the understanding of the putative nephroprotective effects of vitamin C in a pathological psoriasis environment.

## 2 Materials and methods

### 2.1 Animals

Male mice of the C57BL/6 strain (9–11 weeks old, weight range 25–30 g) were from the Bioterium of The Institute of Neurobiology, UNAM, Queretaro, Mexico. Mice were placed in cages (7 mice per cage) at 23–25 °C under a 12/12 light-dark cycle and were left for seven days for adaptation with free access to food and water. Experiments were conducted following the National Institutes of Health Guide for the Care and Use of Laboratory Animals [33], the ARRIVE guidelines [34], the Mexican regulations regarding the use of experimental animals [35], and approved by the Mexican National Council on Humanities, Sciences, and Technologies (CONAHCYT ID#:3969863). Mice were sacrificed by intraperitoneal injection of sodium pentobarbital (PiSA, Hidalgo, Mexico, 65 ml/Kg b.w.).

### 2.2 IMQ-induced psoriasis and MTX-triggered nephrotoxicity

MTX-triggered nephrotoxicity in psoriasis was assessed by establishing the MTX-driven renal injury in an IMQ-induced psoriasis-like mouse model. This model is a powerful tool to study psoriasis as it shows a similarity with human psoriasis regarding: a) the dependence of the IL-23/17 axis, b) the role of IL-22 in psoriasis development, c) the impact of IL-36 in neutrophil recruitment, d) the presence of skin erythema, thickening, scaling, acanthosis and parakeratosis; e) the composition of the inflammatory infiltrate [36].

Animals were treated with Aldara® cream (Meda AB, Solna, Sweden) containing 5 % IMQ (3.125 mg) on their shaved back every 24 h for seven days (n = 4) as previously described [32]. For nephrotoxicity induction, a high-MTX single dose of 20 mg/kg i.p was used and administered on the sixth day, six hours after IMQ application. This dose has been used previously to provoke MTX-mediated acute nephrotoxicity in rodents [25], [37], [38], [39] and corresponds to a human equivalent MTX dose of 1.6 mg/kg [40]. It is close to doses used for psoriasis comorbidities such as cancer [41], [42] and could be representative of MTX overdoses. VitC (175 mg/kg/day) was administered every day for seven days before MTX administration. This approach was chosen based on previous evidence suggesting that pretreatment with vitC before using pro-oxidative agents effectively restores oxidative balance [43], [44]*.* Doses of vitC between 50 and 250 mg/kg have been demonstrated to reduce toxic MTX-mediated effects in rodents [30], [31], [45].

The study groups were as follows: (n=4): group 1 (negative control without treatment); group 2 (positive control-IMQ-induced psoriasis); group 3 (IMQ + vitC 175 mg/kg/day); group 4 (MTX 20 mg/kg i.p); group 5 (IMQ + MTX 20 mg/kg); group 6 (IMQ + vitC 175 mg/kg/day + MTX 20 mg/kg). Weight and food consumption were monitored during the seven days of the trial.

### 2.3 Psoriasis severity and histopathology

The Psoriasis Area and Severity Index (PASI) score was determined to assess the severity of IMQ-induced psoriasis, considering the evolution of erythema, peeling, and thickening according to a calculator (www.pasi.corti) as previously reported [46]. A cardiac puncture was performed to obtain blood samples, while for histopathological analysis, kidneys were preserved in a 4 % formaldehyde solution (Hycel, Zapopan, Mexico) in a phosphate-buffered solution (PBS). The kidneys were then dehydrated with a graded ethanol series, cleared in dimethylbenzene, and embedded in paraffin (Leica Biosystems, Nussloch, Germany). Hematoxylin and eosin (H&E) staining was performed according to standard procedures. The histopathological analysis was performed by two independent pathologists from Universidad Michoacana, Mexico, and this analysis was blinded for both pathologists.

### 2.4 Kidney homogenates and protein determination

The kidney sections were homogenized in an ice bath with a phosphate buffer (1:10 w/v) for ten seconds. Tissues were then centrifuged at 4000 rpm for 15 minutes to obtain the supernatant, which was stored at −70°C for subsequent analysis. Protein quantification of each sample was performed in duplicate using the Bradford assay [47].

### 2.5 Serum creatinine and kidney inflammation

Serum creatinine was determined using the Jaffé method. Creatinine reacted with alkaline picrate, forming a reddish complex whose intensity is associated with the creatinine concentration [48]. The TNF-α production was quantified in kidney homogenates using a commercial LegendMax ELISA kit and following the manufacturer's instructions to determine the impact of treatments on renal inflammation (430907; BioLegend®, San Diego, USA).

### 2.6 Assessment of renal oxidative stress and antioxidant markers

Considering that MTX-induced renal damage has been connected to OS [17], [18], several markers related to oxidative stress (malondialdehyde, 8-hydroxydeoxyguanosine, nitrites/nitrates) and antioxidant defense (superoxide dismutase, total antioxidant capacity) were determined in kidney homogenates. They were selected considering previous studies reporting their alterations because of MTX-induced toxicity [24], [25], [27], [31], [49]. Moreover, they cover different aspects of oxidative damage and antioxidant defense mechanisms and have already been used to determine the oxidative status in psoriatic patients [50]*.*

#### 2.6.1 Malondialdehyde (MDA)

The thiobarbituric acid-reactive substance (TBARS) assay kit (Cayman Chemical Company®, 10009055, Ann Arbor, Michigan, USA) determined the MDA levels. Kidney homogenates (100 µl) were mixed with 100 µl sodium dodecyl sulfate (SDS) and 800 µl of thiobarbituric acid; then the mixture was heated for one hour. Subsequently, the tubes were placed on ice to stop the reaction. The tubes were then centrifuged at 1600 x g, and 200 microliters of the supernatant were transferred to a microplate. Both samples and MDA standards (0.625–50 µM) were run in duplicate. The absorbance was read at 540 nm.

#### 2.6.2 Nitrites and nitrates

The levels of nitrates and nitrites in kidney homogenates were determined using the nitrite/nitrate assay kit according to the manufacturer's methodology (Sigma-Aldrich ®, 23479, Toluca, MX). The assay started by adding 10 µL of the nitrate reductase solution and 10 µL of the enzyme co-factors solution to the wells designated for [NO_3_^-^ + NO_2_^-^] detection, including kidney homogenates and standard wells (80 µl). Then, the content was thoroughly mixed in a horizontal shaker, and the plate was incubated at 25 °C for two hours. Subsequently, 50 µL of Griess Reagent A was added to each well. The mixture was then stirred using the shaker and incubated at 25 °C for five minutes. The plate was incubated at 25 °C for an additional 10 min. Both samples and standards were analyzed in duplicate. The absorbance was read at 540 nm.

#### 2.6.3 Superoxide dismutase (SOD)

The enzymatic activity of SOD was determined using the SOD assay kit according to the manufacturer's instructions (Cayman Chemical Company®, 706002, Ann Arbor, Michigan, USA). Briefly, 200 μL of the radicals’ detector containing tetrazolium salt solution was mixed with 10 μL of the kidney homogenates or SOD standard. Then, 20 μL of xanthine oxidase was added into the wells, incubated on a shaker for 20 minutes at room temperature, and absorbance measured at 440 nm. The SOD activity was calculated from a standard curve and expressed as U/ml.

#### 2.6.4 Total antioxidant capacity (TAC)

TAC analysis was carried out using the antioxidant assay kit following the manufacturer's directions (Sigma-Aldrich, CS0790, St.Louis, MO, USA). For this, 20 µL microliters of the kidney homogenates and 10 µL of myoglobin were mixed in a 96-well plate. Subsequently, 150 µL of ABTS solution was added to each well, and the mixture was incubated at room temperature on a shaker for five minutes. Following incubation, 100 µL of stop solution was added, and absorbance was read at 450 nm. The results were compared to a Trolox standard curve (0.015–0.42 mM). All measurements were conducted in duplicate.

#### 2.6.5 Oxidative DNA Damage (8-OHdG)

According to the manufacturer's instructions, the oxidative DNA damage was determined using an 8-hydroxy-2-deoxyguanosine (8-OHdG) ELISA kit (MyBioSource®, MBS267161, San Diego, USA). 50 μL of kidney homogenates or an 8-OHdG standard were added to each well and mixed with horseradish peroxidase (HRP) conjugated 8-OHdG antibody. A tetramethylbenzidine (TMB) substrate was used to obtain a color signal at 450 nm. The 8-OHdG concentration of kidney homogenates was determined using a standard curve.

### 2.7 Statistical analysis

All values were expressed as mean ± standard error (SEM). The GraphPad Prism software (version 8) was used to compare the effect of different treatments using unpaired two-tailed Student's t-tests or one-way ANOVA followed by Tukey's post hoc comparison tests. A p=<0.05 was considered statistically significant.

## 3 Results

### 3.1 Body weight and food intake

**Fig. 1:**
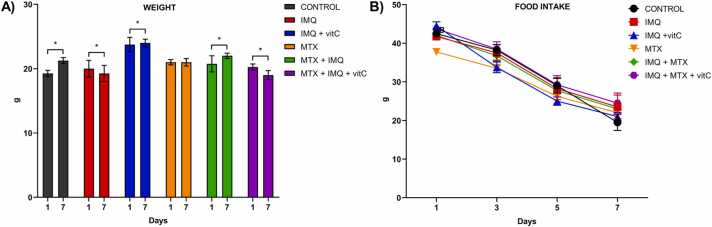
Evolution of body weight gain (A) and food consumption (B) in experimental groups. Results represent mean ± SEM.*p< 0.05 Student t-test. C57BL/6 Mice received IMQ topically (3.125 mg) on their shaved back every 24 h for seven days. For nephrotoxicity induction, methotrexate (MTX) was administered in a single dose of 20 mg/kg/b.w i.p. Mice received vitamin C (vitC, 175 mg/kg/b.w i.p) with IMQ or MTX every day for seven days.

Fig. 1 represents the evolution of body weight and food consumption in the experimental groups. Control and mice that received IMQ+MTX gained weight throughout the experiment, while the groups that received IMQ and IMQ+MTX+VitC showed a decrease in their body weight. Groups that received IMQ+vitC and those administered with MTX maintained a constant weight. Although variations in body weight were recorded, food consumption decreased during the experiment in all groups with no statistically significant differences between the treatments (p>0.05).

### 3.2 Psoriasis severity

**Fig. 2:**
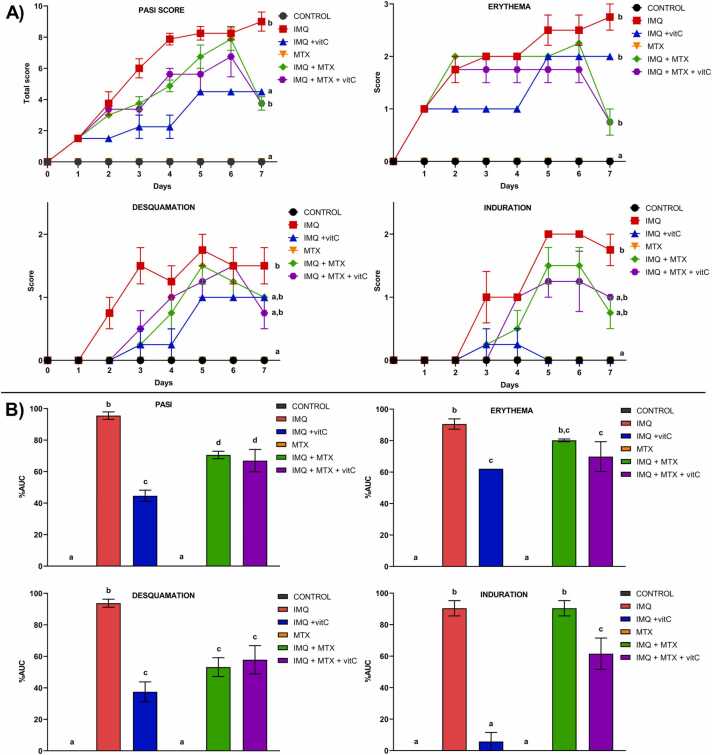
Psoriasis severity under different treatments. A) Temporal evolution of the clinical psoriasis features; B) Treatment effects. Results are shown as mean ± SEM. Different letters over columns indicate statistically significant differences between groups. ANOVA and post hoc Tukey test **(p< 0.05).** AUC (area under the curve); MTX (Methotrexate); IMQ (Imiquimod); vitC (Vitamin C).

Fig. 2 shows the evolution over time of the clinical features of psoriasis and the effect of the different treatments. According to the PASI score, animals that received IMQ on days 2–7 displayed signs of scaling, erythema, and thickening characteristics of psoriasis skin (PASI=9 at the end of the experiment corresponding to moderate psoriasis). vitC significantly decreased erythema, desquamation, and induration. These effects were mainly observed after the second day after IMQ application, while mice administered with IMQ+MTX notably decreased desquamation compared to the IMQ group (p<0.05). These effects were statistically significant after the sixth day after the IMQ application. Moreover, the group that received IMQ+MTX+vitC significantly decreased the induration compared to the IMQ+MTX group (p<0.05). However, neither erythema nor desquamation were significantly modified by treatment with IMQ+MTX+vitC compared to mice administered with IMQ+MTX.

Overall, the evaluation of the PASI score showed a significant decrease in psoriasis severity in the IMQ+vitC, IMQ+MTX, and IMQ+MTX+vitC groups *vs*. the IMQ (p<0.05). However, mice that received IMQ+vitC exhibited the most significant effect in reducing the PASI score compared to those administered with IMQ+MTX and IMQ+MTX+vitC (p<0.05).

### 3.3 Histopathological analysis

**Fig. 3:**
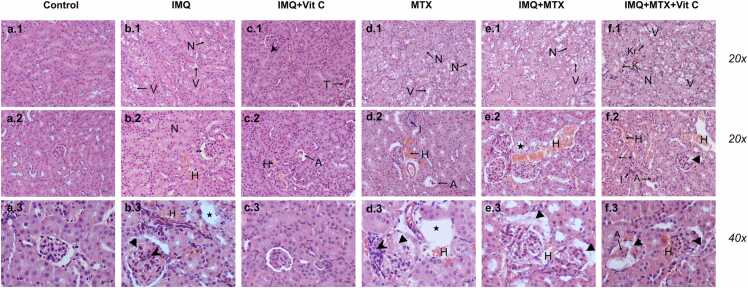
Representative photomicrographs of histopathological sections of kidney tissue. H&E, 20 and 40x. (N)-Acute tubular necrosis, (Star)-Tubule loss, (K)-Karyolysis, (Kr)-Karyorrhexis, (*)-Tubular cells with eosinophilia, (V)-Vacuolization (hydropic degeneration), (I)-Peritubular inflammatory infiltrate, (H)-Interstitial hemorrhage, (Double arrowhead)-Dilatation of Bowman's capsule, (T)-Thrombosis of glomerular capillaries, (Arrowhead)-Mononuclear inflammatory infiltrate, (A)-Glomerular atrophy, (Triangle)-Glomerulosclerosis.

Histological microphotographs of renal tissue under different treatments are shown in Fig. 3. The control group exhibited normal renal histomorphology in the cortex and medulla without alterations in the glomeruli and tubules (Fig. 3**.** a1, a2, a3). Imiquimod application induced renal tubular lesions, characterized by the presence of hydropic vacuoles (hydropic degeneration) with moderate acute tubular necrosis and minor interstitial hemorrhage between the proximal and distal convoluted tubules. In addition, some areas with loss of renal tubules were observed. Bowman's capsule's slight dilation and capillary network congestion were detected at the glomeruli, whereas some glomeruli presented segmental sclerosis and invasion of mononuclear infiltrate (Fig. 3**.** b1, b2, b3).

Interestingly, treatment with vitC improved renal histological lesions induced by IMQ (Fig. 3. c1, c2, c3). Hydropic degeneration in the renal tubules disappeared, peritubular hemorrhage decreased, and glomerular injury improved. However, a few glomerular atrophies and mild tubular necrosis could still be detected in microphotographs.

The MTX administration provoked higher kidney injury than the IMQ application (Fig. 3**.** d1, d2, d3), mainly on tubular cells. This injury was characterized by membrane rupture, cellular swelling, and karyolysis (loss of the nucleus). The increase in hydropic degeneration and acute tubular necrosis was related to irreversible renal damage. Moreover, slight interstitial hemorrhage between the tubules, slight glomerular atrophy, and mild-to-moderate glomerulosclerosis were found in microphotographs.

Acute tubular necrosis was intensified in mice that received IMQ+MTX, compared to those that received MTX or IMQ alone (Fig. 3**.** e1, e2, e3). Consequently, the proximal and distal tubules were affected at the renal cortex and medulla. This damage was accompanied by vacuolar degeneration and karyolysis. The peritubular hemorrhage was mild to moderate, with scarce mononuclear inflammatory infiltrate between the tubules. Glomerular sclerosis increased from moderate to abundant compared to the groups administered with IMQ or MTX.

The acute tubular necrosis was intensified in the IMQ+MTX+vitC group compared to that described in IMQ+MTX, with an increase in eosinophilic cells, hydropic degeneration, cells with karyorrhexis, karyolysis, and detachment of necrotic cells towards the tubular lumen (Fig. 3. f1, f2, f3). Abundant peritubular bleeding was observed in both the cortex and the medulla with mild multifocal inflammation composed of mononuclear infiltrate. An increase of sclerosed glomeruli accompanied by atrophy was also detected in microphotographs. Furthermore, some glomeruli showed extensive capillary thrombosis and periglomerular bleeding.

### 3.4 Serum creatinine and TNF production

**Fig. 4:**
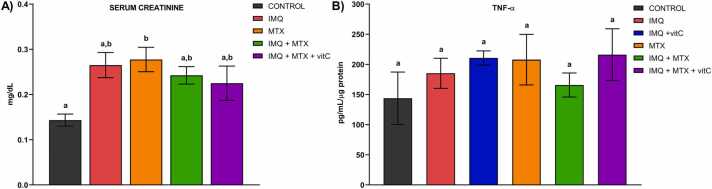
Impact of treatments on serum creatinine (A) and TNF production (B) in kidney homogenates. Results are shown as mean ± SEM. Different letters over columns indicate statistically significant differences between groups. ANOVA and post hoc Tukey test **(p< 0.05).**

Fig. 4 A represents the serum creatinine levels of experimental groups treated with MTX, IMQ, or vitC. As observed, mice that received MTX showed a significant increase in creatinine levels *vs*. the control (p<0.05), whereas those that received IMQ, IMQ+MTX, and IMQ+MTX+VitC showed a trend towards increased creatinine levels compared to the control. In contrast, no significant differences (p>0.05) were found regarding TNF-α levels in kidney homogenates in the experimental groups *vs*. the control (Fig. 4B).

### 3.5 Renal OS and antioxidant markers

**Fig. 5:**
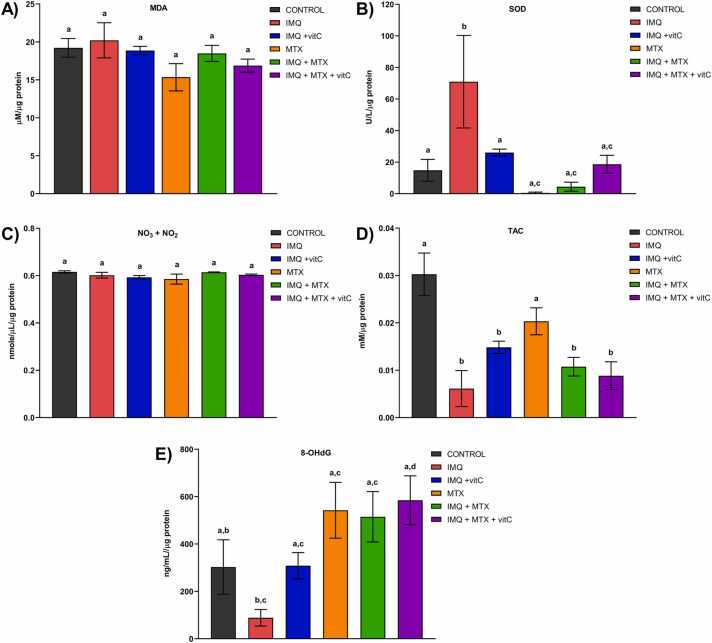
Effects of treatments on renal oxidative stress markers. Malondialdehyde (MDA); B) Superoxide dismutase (SOD); C) Nitrites and nitrates (NO_3_+NO_2_); D) Total antioxidant capacity (TAC); E) 8-hydroxy-2-deoxyguanosine (8OHdG). Results are shown as mean ± SEM. Different letters over columns indicate statistically significant differences between groups. ANOVA and post hoc Tukey test **(p< 0.05).**

The effects of treatments on renal OS and antioxidant markers are represented in Fig. 5. No significant differences were found between the study groups for MDA levels (Fig. 5 **A**). SOD enzymatic activity significantly increased in the IMQ group *vs*. the control (p<0.05), while experimental groups that received IMQ+vitC, MTX+IMQ+vitC, MTX, and IMQ+MTX showed no significant differences *vs.* the control (p>0.05). Mice administered with MTX and MTX+IMQ showed a trend toward decreased SOD activity **(**Fig. 5 **B)**. No significant differences in the experimental groups and control were observed in determining nitrates and nitrites (Fig. 5 **C**). On the other hand, mice administered with IMQ, IMQ+vitC, IMQ+MTX, and IMQ+MTX+vitC significantly decreased the total antioxidant capacity *vs.* the control (p<0.05) (Fig. 5 **D**). A significant increase of DNA damage marker (8-OHdG) was observed in mice administered with IMQ+MTX+vitC *vs.* the IMQ group (p<0.05). Although the other experimental groups were not statistically different *vs*. the control (p>0.05), a trend towards a decrease in this marker *vs.* the control was seen in mice administered with IMQ. In contrast, mice that received MTX, IMQ+MTX, and IMQ+MTX+vitC tended to increase 8-OHdG values *vs*. the control group (Fig. 5 **E**).

## 4 Discussion

Psoriasis is an incurable inflammatory dermatological disease whose etiology is not completely elucidated [1]. Some animal models can emulate its clinical features, including the IMQ-induced psoriasis model, in which mice's skin shows some parallelisms with human psoriasis regarding cytokine pathways, epidermal thickening, cellular infiltration, and the impact of the IL-17/23 axis [32]. This model has been used to investigate the effects of putative antipsoriatic treatments and the influence of OS and inflammation on disease development and perpetuation [32], [51].

MTX is the most prescribed drug for systemic psoriasis treatment [52]. However, long-term use of this drug or high MTX exposition during accidental overdosing or in co-morbid cancer patients [10], [14] can cause nephrotoxicity.

As psoriasis is characterized by systemic inflammation, OS, chronic inflammation, and vascular dysfunction [22], [23], the use of nephrotoxic drugs such as MTX could promote the exacerbation of kidney injury [53], while the use of antioxidants commercially available as vitC could be an appropriate strategy to counteract such side effect.

The PASI is a well-established tool for determining the degree of psoriasis severity. The results show that IMQ treatment increases the erythema (redness), desquamation (scale), and induration (thickness) of mice skin, as previously reported [32]. These signs gradually increased from the second day after the IMQ application, as formerly described [36]. IMQ is a well-known ligand for toll-like receptor (TLR-7/8) impacting plasmacytoid dendritic cells (pDCs), which express constitutively high levels of TLR7. The activation of TLR7 induces pDC infiltration into mouse skin, leading to the production of INF-α and TNF-α required for the IL-23/Th17 pathways activation [54].

Psoriasis severity decreased in the IMQ+MTX and IMQ+MTX+vitC groups compared to the IMQ. This effect was more pronounced after the sixth day of the IMQ application, likely due to the MTX administration [55], [56]. Interestingly, mice treated with IMQ+vitC exhibited a significantly lower PASI score than those of the IMQ, IMQ+MTX, and IMQ+MTX+vitC groups. It suggests that vitamin C may be favorable in attenuating inflammation, erythema, and scaling associated with psoriasis. However, its concurrent use with MTX appears to diminish these protective effects. Previous studies have reported that vitC levels are significantly lower in patients with active psoriasis compared to healthy controls, and a negative correlation has been observed between vitC levels and PASI scores [57]. Thus, vitamin C supplementation has been suggested as a therapeutic approach for improving psoriasis symptoms [57]. Beyond its well-established antioxidant properties, vitamin C exerts anti-inflammatory effects by inhibiting the activity of nuclear factor kappa B (NF-κB), a crucial regulator of the inflammatory response [58]. These characteristics could be related to its effects in improving psoriasis skin lesions induced by IMQ.

These findings are consistent with kidney histology, where vitC improved IMQ-induced renal lesions, including hydropic renal tubular degeneration, peritubular hemorrhage, and glomerular injury. Topical IMQ application can induce acute tubular necrosis in humans [59] and renal dysfunction in mice [60] through activation of the TLR/NF-κB signaling pathway [61]. Furthermore, MTX administration resulted in more severe tubular injury than IMQ alone, which aligns with the well-known nephrotoxic properties of this drug [16], [62]. Notably, acute tubular necrosis increased in mice treated with the IMQ+MTX compared to those receiving either agent individually. This observation suggests that the renal pathological environment characteristic of psoriasis may heighten the sensitivity of renal cells to MTX exposure.

Contrary to expectations, acute tubular necrosis was more severe in the IMQ+MTX+vitC group compared to the IMQ+MTX group, while the IMQ+vitC group exhibited reduced kidney injury relative to the IMQ group. Although previous studies have demonstrated that vitC administration mitigates MTX-induced OS and renal damage [31], [63], the protective effects of vitC on renal toxicity appear to be diminished in a more complex pathological context involving the psoriasis-like inflammation induced by IMQ and the nephrotoxicity elicited by MTX.

Serum creatinine is a primary biochemical marker for assessing kidney function [64]. An elevation in serum creatinine is indicative of reduced excretory capacity and structural renal damage [65]. The present study observed a significant increase in serum creatinine in the MTX group. Moreover, mice treated with IMQ, MTX, and vitC showed a trend toward higher creatinine levels vs. control, thus ratifying renal dysfunction. These findings align with the histopathological evidence of renal impairment.

Previous studies have linked MTX-induced kidney injury to alterations in cytokine production, particularly TNF-α, which is known to promote pro-inflammatory oxidative cell death [27], [66]. However, in the context of nephrotoxicity induced by the administration of IMQ and MTX, this mechanism appears less relevant, as no statistically significant differences were observed in TNF-α levels in kidney homogenates across the experimental and control groups.

In the present study, acute tubular necrosis was the predominant renal damage observed in histological microphotographs, aligning with previous research documenting extensive tubular necrosis in patients treated with MTX [67], [68]. Previous studies have indicated increased dermal hypervascularity and angiogenesis starting from day four following IMQ topical administration in mice [36]. This increase in dermal vascularity could be associated with higher systemic exposure to IMQ and consequent renal toxicity beyond the fourth day of administration. Such a pathological state in the kidneys may lead to elevated and sustained plasmatic MTX levels, thereby exacerbating renal toxicity through a direct toxic effect on the renal tubules, as previously described [62]. This mechanism is characterized by disruptions in cell volume, leading to notable cell swelling, which was evident in renal histology. MTX induces activation of the Na^+^/H^+^ antiporter, resulting in increased sodium influx and proton efflux [62]. In this context, the contribution of oxidative pro-inflammatory cell death driven by TNF-α [27], [68] may be less significant in explaining MTX-induced nephrotoxicity. Nonetheless, further research is needed to investigate this hypothesis [27], [66].

OS has been suggested as a relevant factor in MTX-induced kidney toxicity [24], [25], [26], [27]. Lipid peroxidation, which arises from the interaction between reactive oxygen species and polyunsaturated fatty acids in cell membranes, leads to malondialdehyde (MDA) formation, a well-established marker of OS [69]. Although antioxidants such as quercetin, diosmin, curcumin, and vitamin C have been reported to reduce MDA levels in kidney tissue following MTX administration [31], [39], [70], no significant differences were found in MDA levels among the experimental groups. This finding suggests that MTX-induced renal toxicity in the context of IMQ-induced psoriasis is likely not dependent on lipid peroxidation.

Nitric oxide (NO) plays diverse physiological roles; at low concentrations, it contributes to vasodilation and homeostasis, whereas elevated NO levels are associated with peroxynitrite radicals’ production and subsequent lipid peroxidation. Due to the short half-life of NO, direct measurement is challenging; therefore, its metabolites, nitrates, and nitrites, are commonly utilized to estimate NO production [71]. Although previous studies have shown that MTX administration increases nitric oxide levels in kidney tissue [70], [72], our findings differ, as NO concentrations were comparable across the study groups. Preceding research demonstrated that Infliximab, a well-known TNF-α inhibitor, reduces renal NO levels induced by MTX [73], underscoring the role of TNF-α in elevating renal NO levels through the stimulation of inducible nitric oxide synthase [73], [74]. Consequently, the absence of statistically significant differences in TNF-α production among the experimental groups compared to the control may be related to the observed NO levels. Nonetheless, additional research is required to elucidate the influence of TNF-α on NO production within the context of MTX-induced nephrotoxicity in psoriasis.

SOD is an antioxidant enzyme that converts superoxide radicals into hydrogen peroxide, serving as a primary defense mechanism against ROS [75]. Given the high energy demands of the kidneys, SOD is crucial for maintaining oxidative balance [76]. In the present study, SOD activity increased in the IMQ group compared to control, suggesting a protective kidney response to IMQ-induced nephrotoxicity. Previous studies have indicated that increased SOD activity correlates with renal protection against damage caused by hyperglycemia [77] or hypoxia [78]. Furthermore, the IMQ application for seven days is likely too short to decrease renal SOD activity significantly. Although most of the studies have demonstrated a reduction in SOD activity induced by IMQ in the skin [79], [80], only a few studies have addressed the impact of IMQ on SOD activity in kidneys, and these studies have been performed in Balb/C mice [61], [81]. Evidence supports the strain-dependence regarding psoriasis features and inflammatory biomarkers behavior driven by IMQ in mice, with the C57BL/6 strain being the most consistent model replicating human psoriasis [82]. Genetic differences between mouse strains could influence the expression of renal oxidative stress biomarkers, thereby accounting for the discrepancies regarding renal SOD activity with previous reports [61], [81]. Conversely, in the IMQ+vitC group, renal SOD activity was normalized, showing no significant differences vs. the control, thereby demonstrating a protective vitC effect to counteract the increase in SOD activity induced by IMQ.

Notably, the MTX-treated group exhibited a reduction in SOD activity, consistent with former reports demonstrating decreased renal SOD activity following MTX exposure [26], [31], [70]. Mice receiving MTX+IMQ also showed a similar decrease in SOD activity. However, those administered with IMQ+MTX+vitC displayed a trend toward restoring SOD activity to baseline levels, suggesting that vitC may help mitigate the substantial decrease in SOD activity caused by MTX.

TAC measures the overall antioxidant defenses in the kidneys by integrating the actions of various antioxidants against OS [83]. In the present study, IMQ administration significantly reduced TAC compared to the control group. Conversely, mice treated with IMQ+vitC showed a trend toward increased TAC values, suggesting a potential therapeutic effect of vitC in counteracting pro-oxidative effects driven by IMQ. Mice treated with MTX also exhibited a trend toward reduced TAC compared to controls, consistent with previous studies [84], [85]. Interestingly, the IMQ+MTX+vitC group did not show significant protective effects against the reduction in TAC, as TAC values were like those found in the IMQ+MTX group. These findings indicate that while vitC may protect against reductions in renal TAC during IMQ-induced psoriasis, this protective effect appears compromised when vitC is administered with IMQ and MTX.

8-OHdG is a marker of DNA oxidative damage whose elevated levels have been associated with increased mortality risk in chronic kidney disease [86] and renal fibrosis [87]. In this study, mice treated with IMQ exhibited a trend toward reduced 8-OHdG levels compared to controls, which may reflect an adaptive response by kidney cells to mitigate DNA damage induced by IMQ. Imiquimod, a TLR7/8 agonist, has been shown to enhance the expression and nuclear localization of DNA repair genes in a MyD88-dependent manner in bone marrow-derived cells [88]. Additionally, skin antigen-presenting cells stimulated by IMQ demonstrate effective DNA repair mechanisms against ionizing and non-ionizing radiation [88]. Interestingly, the observed decrease in 8-OHdG levels appears to be negatively correlated with the increased SOD activity induced by IMQ. Preceding research indicates that elevated SOD activity can mitigate DNA damage and reduce 8-OHdG levels in response to xenobiotic exposure [89], [90]. Mice administered with IMQ+vitC restored 8-OHdG levels like the control group, suggesting that vitC may help normalize DNA oxidative damage.

Conversely, groups treated with MTX, IMQ+MTX, and IMQ+MTX+vitC exhibited a trend toward increased 8-OHdG levels compared to controls. Previous studies have documented that MTX administration raises 8-OHdG levels in various tissues, including the lung [91], liver [92], and kidney [49]. In this context, vitC did not mitigate the oxidative DNA damage induced by MTX.

Overall, the results of this study provide new insights into MTX-induced acute nephrotoxicity considering the context of psoriasis and the potential mitigating effects of vitC. The data indicate that the combination of IMQ and MTX exacerbates nephrotoxicity compared to MTX alone, evidenced by increased acute tubular necrosis and peritubular hemorrhage, along with a significant reduction in the total antioxidant capacity of renal cells. Given that IMQ induced mild renal lesions and MTX was administered six days after the start of IMQ application, it is plausible that the initial renal damage caused by IMQ compromised the clearance of MTX, thereby amplifying its nephrotoxic effects. It highlights the complex interplay between IMQ-induced renal impairment and MTX acute toxicity, suggesting that the timing and combination of these treatments can significantly impact renal outcomes.

This study also shows the differences in the vitC effects depending on the pathological context. Vitamin C improved the clinical skin psoriasis lesions and attenuated renal lesions induced by IMQ. Consequently, consuming diets rich in vitamin C or ingesting dietary vitamin C supplements could be a strategy to treat psoriasis patients with compromised renal function. Although the mechanism of action of vitamin C in psoriasis has not been fully elucidated, the results of this research suggest that these effects would probably be related to its antioxidant activity since its concomitant use with IMQ improved total renal antioxidant capacity and restored SOD activity and 8-OHdG levels.

On the contrary, when vitamin C is administered in the context of renal impairment triggered by IMQ and MTX, these protective effects are reversed. Although there is no consensus regarding the impact of vitamin C on urine acidification, some research has shown that it causes significant reductions in urinary pH [93], [94]. At pH below 7, the solubility of high MTX doses may be surpassed, leading to intratubular crystal formation and renal impairment [95]. Thus, the deterioration of renal function caused by IMQ and the consequent poor MTX elimination, coupled with its low solubility at acidic pH, could contribute to MTX crystallization, thereby exacerbating the tubular injury. Further research is warranted to verify this hypothesis and identify the signaling pathways involved in MTX-triggered acute tubular necrosis in psoriasis.

## 5 Conclusions

This study is the first to explore acute nephrotoxicity induced by MTX and the impact of vitC in an IMQ-induced psoriasis-like mouse model, distinguishing it from prior research that examined vitC's effects on MTX toxicity in animals without additional pathology. Since psoriasis patients commonly use MTX and vitC, this model provides a more realistic scenario to assess their combined acute toxicological effects. However, the lack of chronicity of the IMQ-induced psoriasis model [32] limits the translatability of these findings to MTX-induced nephrotoxicity during long-term treatments.

While vitamin C protects against IMQ-induced renal damage, its efficacy decreases when administered alongside MTX in psoriasis. Since kidney diseases often accompany psoriasis, and MTX remains a crucial treatment, our findings suggest that vitamin C supplementation may not be advisable for psoriatic patients with renal impairment undergoing MTX therapy at high doses. Future research should focus on elucidating the signaling pathways involved in the vitamin C effects tested at different doses during long-term antipsoriatic treatments with MTX.

## CRediT authorship contribution statement

**Martha-Estrella García-Pérez:** Writing – review & editing, Supervision, Resources, Project administration, Methodology, Formal analysis, Conceptualization. **Sandra-Guadalupe Sánchez-Ceja:** Writing – review & editing, Supervision, Methodology, Investigation, Formal analysis. **Grecia-Elena Hurtado-Nuñez:** Writing – review & editing, Supervision, Methodology, Investigation, Formal analysis. **Elodia-Nataly Díaz-De-la-Cruz:** Writing – review & editing, Writing – original draft, Visualization, Software, Methodology, Investigation, Formal analysis. **Walter-Ángel Trujillo-Rangel:** Writing – review & editing, Supervision, Resources, Methodology, Investigation, Formal analysis, Conceptualization. **María-Carmen Bartolomé-Camacho:** Writing – review & editing, Supervision, Methodology. **Luz Torner:** Writing – review & editing, Supervision, Methodology, Investigation.

## Declaration of Competing Interest

The authors declare the following financial interests/personal relationships which may be considered as potential competing interests: Elodia Nataly Diaz de la Cruz reports financial support was provided by National Council on Science and Technology (CONAHCYT). If there are other authors, they declare that they have no known competing financial interests or personal relationships that could have appeared to influence the work reported in this paper.

## Data Availability

Data will be made available on request.
